# The Influenza Epidemic in a Camp

**Published:** 1918-11

**Authors:** 


**The Influenza Epidemic in a Camp.** By C. Averill, G. Young and J. Griffiths. *The British Medical Journal,* August 3, 1918.

Most of the cases were of characteristic type, admitted with temperature ranging from 101 to 104 degrees, complaining of headache, backache, general aching, and sore throat. The majority stated that they had been suddenly taken ill, with a feeling of great weakness. Apart from the fever, some congestion of the pharynx, and in a few cases slight bronchial catarrah, they presented no other symptoms.

The illness ran a short course, the average duration of stay in hospital being 5 days. Within 24 to 48 hours of admission the temperature dropped by crisis to normal, or hovered between 99° and 103° for a few days.

Complications were few. Several patients had obstinate constipation; one or two had mild diarrhea. Cough in a few cases was troublesome. Although the incidence of pneumonia was low, the case death rate for pneumonia was high.

*Preventive Measures* : The whole of the contacts of three units were passed through the Levick spraying chamber. No reduction occurred in the number of admissions from these units, there being a greater incidence after than before the use of this method. Gargling with permanganate solution (1 in 5000) was tried, but no effect was noticed.

Spraying the nose and throat with Acro-flavine solution (1 in 1000) seemed to have a distinct preventive effect.

*Bacteriology* : Bacteriological investigations comprised the examination of (1) smears and growth from naso-pharynx; (2) smears and growth from sputum; (3) blood cultures; (4) growth from cerebrospinal fluid.

1. *Nasopharynx.* (*a*) Smears : there was a predominance of a Gram-positive diplococcus. Pfeiffer's bacillus absent. (*b*) Culture : Gram-positive diplococcus. Pfeiffer's bacillus absent.

2. *Sputum.* (*a*) Smears : Gram-negative bacilli and Gram-positive diplococci were found. (*b*) Culture : the media used were agar, trypsin blood agar with rabbits, blood enrichment and trypsin agar with human blood. The same sputa were used. The two organisms found in the sputum were in every respect identical with the Pfeiffer's bacillus and the pneumococcus.

3. *Blood.* Nine cases with marked symptoms were chosen for blood cultures. Of these all were sterile.

